# Supplementary material for: SLC6A4 DNA Methylation Levels and Serum Kynurenine/Tryptophan Ratio in Eating Disorders: A Possible Link with Psychopathological Traits?
Source: Nutrients. 2023 Jan 13;15(2):406. doi: 10.3390/nu15020406 (PMC9866524; doi:10.3390/nu15020406)
Supplement: Supplementary file 1 [file nutrients-15-00406-s001.zip › nutrients-2127265-supplementary.pdf]

Supplementary table

SLC6A4 DNA methylation levels and serum kynurenine/tryptophan ratio in Eating Disorders: a possible link with psychopathological traits?

Marica Franzago[1,2], Elena Orecchini[3], Annamaria Porreca[4], Giada Mondanelli[3], Ciriana Orabona[3], Laura Dalla Ragione[5], Marta Di Nicola[4], Liborio Stuppia[2,6], Ester Vitacolonna[1,2], Tommaso Beccari[7], Maria Rachele Ceccarini[7*]

[1]Department of Medicine and Aging, School of Medicine and Health Sciences, "G. d'Annunzio" University, Chieti, Italy, marica.franzago@unich.it, e.vitacolonna@unich.it

[2]Center for Advanced Studies and Technology, "G. d'Annunzio" University, Chieti, Italy, marica.franzago@unich.it, stuppia@unich.it, e.vitacolonna@unich.it

[3]Department of Medicine and Surgery, University of Perugia, Perugia, Italy, elena.orecchini@gmail.com, giada.mondanelli@unipg.it, ciriana.orabona@unipg.it

[4]Laboratory of Biostatistics, Department of Medical, Oral and Biotechnological Sciences, "G. d'Annunzio" University, Chieti, Italy, porreca.annamaria@gmail.com, marta.dinicola@unich.it

[5]Food Science and Human Nutrition Unit, University Campus Biomedico of Rome, Rome, Italy, dallaragione@gmail.com

[6]Department of Psychological, Health and Territorial Sciences, School of Medicine and Health Sciences, "G. d'Annunzio" University, Chieti, Italy, stuppia@unich.it

[7]Department of Pharmaceutical Sciences, University of Perugia, Perugia, Italy, tommaso.beccari@unipg.it, mariarachele.ceccarini@unipg.it

*Correspondence: Maria Rachele Ceccarini, mariarachele.ceccarini@unipg.it, +39 075 585 7905

Supplementary Table S1. Correlations among *SLC6A4* methylation and serum Kynurenine/Tryptophan (Kyn/Trp) levels in EDs patients.

Groups	*Variables*	*SLC6A4 CpG1%*	*SLC6A4 CpG2%*	*SLC6A4 CpG3%*	*SLC6A4 CpG4%*	*SLC6A4 CpG5%*	*SLC6A4 CpG6%*	*Mean Methylation %*
AN0	*Kyn*	0.493*	0.070	-0.140	0.080	0.027	0.245	0.196
	Trp	0.044	-0.053	-0.566**	-0.062	-0.280	-0.313	-0.258
	*Kyn/Trp*1000*	0.418*	0.125	0.297	0.020	0.120	0.428*	0.317
AN1	*Kyn*	0.112	0.210	0.228	0.133	-0.352	0.317	0.154
	Trp	0.287	0.476	0.427	0.280	0.014	0.217	0.462
	*Kyn/Trp*1000*	-0.112	-0.154	-0.123	-0.119	-0.377	0.117	-0.280
BED	*Kyn*	-0.057	0.352	0.153	0.290	0.342	0.471*	0.444*
	Trp	-0.162	0.048	-0.122	0.286	0.459*	0.329	0.296
	*Kyn/Trp*1000*	0.010	0.297	0.184	0.061	0.070	0.232	0.247
BN	*Kyn*	0.357	-0.476	-0.304	-0.024	-0.327	0.109	-0.167
	Trp	0.452	-0.714	0.101	0.214	-0.109	0.327	0.071
	*Kyn/Trp*1000*	0.476	0.595	0.203	0.262	0.518	0.191	0.476
UFED	*Kyn*	-0.083	-0.583	-0.390	0.067	0.441	0.091	-0.233
	Trp	-0.417	-0.117	-0.136	-0.500	-0.153	-0.310	-0.100
	*Kyn/Trp*1000*	0.250	-0.467	-0.220	0.367	0.542	0.347	-0.133
OSFED	*Kyn*	0.152	-0.093	0.287	0.764***	0.220	0.371	0.364
	Trp	-0.108	-0.189	-0.145	0.250	-0.044	0.251	0.059
	*Kyn/Trp*1000*	0.456	0.096	0.482	0.809***	0.267	0.345	0.492*

Computed correlation used the Spearman method with listwise-deletion. Significance code: * p < .05, ** p < .01, *** p < .001. Abbreviations:AN0=restricting anorexia nervosa; AN1= purging anorexia nervosa; BN= bulimia; BED= binge eating disorders; UFED=unspecified feeding or eating disorder; OSFED= other specified eating disorders
